# Oxytocics in Obstetrics

**Published:** 1881-01

**Authors:** S. R. Voorhees

**Affiliations:** Mason, Ohio


### OXYTOCICS IN OBSTETRICS.

By S. R. VOORHEES, M.D., Mason, Ohio.

This is an old subject, but yet it is still in use and will continue to be as long as the present method of propagating the human species is adopted as proper and fashionable. It seems to be in accordance with the American mind to do everything in a hurry, especially when anything comes as near being an unpleasant pastime as parturition. During confinement the patient often solicits the physician to administer something to mitigate her suffering and terminate labor. It often places a physician in a precarious condition, when he has a restless, nervous patient on hand, and a few impatient women, who are constantly whispering and pantomiming to your patient that perhaps the doctor does not understand his business, or is going to do something out of the regular routine that has been followed by his ancestors. Now, in order to facilitate labor, and have everything act in the most favorable manner to all the surroundings, the physician must have the entire confidence of his patient. In order to do this, he must act with that dignity and decorum becoming a physician, and have his patient to understand that he is master of the situation. Then, the best oxytocic is a determined will on the part of the patient to endure the pain and bring all the powers of the mind to bear upon the expulsory efforts of the uterus to expel the fœtus. In my experience, I find that where the patient is restless and not inclined to remain in one position, the best plan is to put her on her left side, limbs fixed, and an assistant holding her hands and supporting her knees, so that she cannot change her position, for sometimes the head will not engage the superior strait when the position of the body is being constantly changed. Having the patient in a proper position, and all things in readiness even then the uterine efforts do not accomplish their object. Here the patient becomes discouraged, and medical aid must be called to your assistance. On examination, the os is not sufficiently dilated to apply the forceps, and some medicine must be administered to assist dilatation and expulsion.

The uterus is supplied with nerves from two different sources; the uterine nerves and ovarian plexus from the great sympathetic; and from the pudic nerve, a branch of the sacral plexus. The ovarian plexus is distributed to the ovaries and fundus of the uterus. The uterine nerves arise from the lower part of the hypogastric plexus, and are distributed to the cervix uteri and the lower part of the uterus. The sympathetic are partially under the control of the will, and something must be done to control the will.

Oxytocics are of two kinds, those which increase muscular action, and those which destroy the sense of feeling. The muscular action may not be strong enough to cause the membranes to press hard enough against the internal os to produce dilatation, or there may be inflammation or ulceration of the os uteri to such an extent that the pain is so severe that it cannot be endured. The pains may be of a neuralgic character, or of a malarial nature.

If I find, on examination, the os closed, pains regular and severe and not of an expulsive nature, I administer about five grains of quinine at once, and if there be a tender os and cervix I add a small opiate—very small, not enough to produce sleep, but enough to quiet the nervous system. If in two hours the nature and condition of things are not changed, I repeat the dose with an additional opiate. But often before the end of two hours the pains may cease, and labor not come on for several days, or the os may dilate rapidly and labor come on naturally. I find quinine good and safe to administer where real labor-pains are coming on, in ordar to stimulate the system to action; but I do not think it will produce labor-pains, or will produce abortion even if given in large doses—only control them where the pains are of a neuralgic nature, or from some other cause aside from parturition. Ergot has the reputation of being an oxyocic, but, in my experience, it acts on the uterine nerves which are distributed to the os cervix, and the lower part of the uterus, causing the lower part of the uterus to contract rather than the fundus, and by so doing retard labor unless the head has eccaped beyond the mouth of the womb.

It seems from looking over the literature on this subject, that nearly every physician has his particular medicine which he gives during parturition. M. Arneville, in *American Journal of Obsterics*, in 1869, says: "On the 24th of last May, I was called by a mid-wife to see a lady, thirty years of age, large, strong, and a primipara. Two hours after the child was delivered, the placenta not having come away, the mid-wife had ministered some ergot of rye, but instead of producing thereby the expulsive pains she had expected, the uterine contractions confined themselves to the mucous fibres of the neck, which closed completely. . . . In two hours the spasm relaxed and the os was supple and sufficiently dilated to admit the end of the hand shaped in a cone." It seems from his statement that ergot acts on the neck and os of the uterus.

Quinine is regarded by many of the profession as a valuable oxytocic in labor, as will be seen from the following quotations taken from the *Half-Yearly Compendium*, July, 1876: "Dr. Paul reports two observations proving its efficiency for exciting the contractions of the fatigued uterus. Dr. Voghera says that quinine given to some women who during pregnancy were attacked with neuralgia, as for other causes, prevoked abortion ; that given to some women at full term of pregnancy it brought on labor ; and that when these women experienced slight irreglar pains, the sulphate of lime rapidly caused the expulsion of the fœtus. It likewise facilitates the expulsion of the placenta; and when in the puerperal state the lochia are suspended, a dose of quinine is sufficient to re-establish it."

Again, other physicians combine morphia, in small doses, with quinine, especially where there is hyperæsthesia of the parts, and report happy results.

Dr. James A. Agnew, of Burkeville, Virginia, writes to *Virginia Medical Monthly*, in November, 1876, about gelsemium in the dilatation of the cervix uteri. In giving ten-drop doses every ten minutes until thirty drops had been given, the cervix dilated rapidly.

Dover's powder has been used in cases of rigidity of the os with good success, where there was irritability and extreme restlessness; but where there are spasms of the uterus, hydrate of chloral, combined with Dover's powder, renders it more efficient. Chloral alone has been given with very good results, where the pains were not bearable and the patient very restless—giving it in full doses every hour or two until delivery is completed. Indeed, I find chloral the safest to give where the case promises to be lingering.

Anæsthetics are advocated by our best authors, and again condemned by others. Where the muscular action of the uterus is strong and the patient determined not to endure the pains, then chloroform must be given. It acts on the spinal plexus of the nerves and obtunds their sensibility.

Harvey L. Byrd, M.D., Professor of Obstetrics in Washington University of Baltimore, Maryland, communicates the following on the action of chloroform in labor, in the

*Baltimore Medical Journal and Bulletin* for February, 1871: "The body of the womb receives its supply from the ganglionic or sympathetic system, while those distributed to the cervix and os are derived from the spinal or nervous centers of animal life. Thus we are enabled to comprehend why it is that ergot, gossypium, etc., as they direct their action to the sympathetic system or nerves of organic life, promote contraction of the muscular fibres of the fundus and body of the uterus, thereby aiding the expulsion of its contents, while morphia, chloroform, etc., obtund in their action the sensibility of the spinal nerves supplying the neck and os, when suffering severely from distention taking place in the fibres of those parts, induced by the pressure of the head or other presenting parts of the fœtus, and thus render less painful, or even painless, the expulsion of the child by the contractions controlled by the ganglonic nerves. The fact is well known to all accouchers of much experience, that *the contractions of the womb are but little influenced by* chloroform, whether the patient is more or less under its influence during labor.

Dr. Haughton, in the *Dublin Medical Quarterly* for May, 1870, says that in the first stage of labor the involuntary muscles of the uterus contract upon the fluid contents of the organ and possess sufficient force to dilate the mouth of the womb and generally rupture the membranes. In the second stage of labor, reflex action calls in the voluntary abdominal muscles, which aid powerfully the uterine muscles in expelling the fœtus. As a consequence, if chloroform is used beyond the step of inducing drunkenness and indifference to pain, it is positively injurious, by cutting off the action of the voluntary muscles, without which the uterus is insufficient. He further states, that the force exerted by the involuntary muscles of the uterus amount to five hundred and twenty-three pounds, and the force of the voluntary muscles is fifty-four pounds, making five hundred and seventy-seven pounds exerted on the child, when all the powers are brought to bear on the child. So, if chloroform paralyzes a part of the force, surgical interferences may have to be brought into requisition before the woman can be delivered.

Dr. W. H. Long, Surgeon U. S. Marine Hospital service, writes to the *Louisville Medical News*, March 16, 1878, as follows: "I desire to call the attention of the profession to the value of viscum album (mistletoe) as an oxytocic. An experience of ten years with this remedy, enables me to speak confidently as to its properties. I was first led to its use by the fact that farmers in the section of country where I formerly practiced medicine gave mistletoe to such of their domestic animals as failed to 'clear' themselves or expel the placenta after bringing forth their young, to promote delivery."

Another article is used with some satisfaction by some practitioners, and that is ustilago maidis, or the fungus product of Indian corn. It produces violent spasmodic contractions of the uterus, and rapid expulsions of its contents. Its use has not been generally adopted by the profession to any great extent.

Alcohol is used to increase the action of the uterine muscles, and it seems to produce good results. It destroys the sensibilitiy of the parts, renders her less indifferent to what is going on, and if the extremities are cold and mind depressed, it increases the circulation and elevates the drooping spirits. But where there is danger of hemorrhage it is not safe to administer it until temulence is produced.

There are other uterine stimulants which are used with great benefit, where the woman becomes impatient and a little time is wanting for the relaxation of the parts and a warming up of the system. These are pepper and ginger tea, hot vaginal injections of water or flax seed or slippery elm tea. The hot water alone removes too much the natural lubricating mucous, which is poured out in great abundance, and thus leaves the parts hot and dry, and so retard labor.

Faradization of the uterus in the later stages of delivery seems to facilitate labor, when the pains are becoming feeble and the woman exhausted. The mode of application consists in placing the positive pole on the lumbar region and the negative immediately below the pubis. The child in no instance seems to have suffered any harm, although by its vigorous movement it clearly showed that it preceived and responded to the excitation of the currents.

